# First record of the myrmicine ant genus *Carebara* Westwood, 1840 (Hymenoptera, Formicidae) from Saudi Arabia with description of a new species, *C. abuhurayri* sp. n.

**DOI:** 10.3897/zookeys.92.770

**Published:** 2011-04-28

**Authors:** Abdulrahman S. Aldawood, Mostafa R. Sharaf, Brian Taylor

**Affiliations:** 1 Plant Protection Department, College of Food and Agriculture Sciences, King Saud University, Riyadh 11451, PO Box 2460, Kingdom of Saudi Arabia; 2 11, Grazingfield, Wilford, Nottingham, NG11 7FN, U.K.

**Keywords:** Ant fauna, Palaearctic, Asir province, Al Bahah, Arabia, new species, Myrmicinae, taxonomy

## Abstract

The myrmicine ant genus *Carebara* is recorded for the first time in Saudi Arabia from the Arabian Peninsula as a whole. A new species *Carebara abuhurayri* **sp. n.** is described based on workers collected from Al Bahah region. One of the smallest ant species known to occur in Arabia, *Carebara abuhurayri* is found in an area inhabited by many ant species including *Tetramorium sericeiventre* Emery, 1877, *Pheidole minuscula* Bernard, 1952, *Pheidole* sp., *Monomorium destructor* (Jerdon, 1851), *Monomorium exiguum* (Forel, 1894) and *Monomorium* sp. and *Crematogaster* sp.

## Introduction

The ant genus *Carebara* Westwood, 1840**,** *sensu*Fernández (2004),is one of the largest ant genera of subfamily Myrmicinae with more than 180 species (Bolton et al. 2006) distributed worldwide in the tropics (Brown 2000) and the Afrotropical region (Weber 1950). Many of them are very tiny cryptic soil and leaf litter inhabitants (Longino 2004). They nest in rotten wood to which the bark is still adherent in the Afrotropical region (Bolton 1973), or may be lestobiotic (Longino 2004) nesting near other ant species. Little is known about the biology of the species.

The taxonomic knowledge also is limited. Fernández (2004) is the most comprehensive study but that dealt primarily with American species. He proposed a significant change to the systematics, however, in arguing for the combination of several genera under the single genus *Carebara*. Thus: *Carebara* Westwood, 1840; = *Oligomyrmex* Mayr, 1867 = *Aeromyrma* Forel, 1891; = *Aneleus* Emery, 1900; = *Erebomyrma* Wheeler, 1903; = *Paedalgus* Forel, 1911; = *Lecanomyrma* Forel, 1913; = *Spelaeomyrmex* Wheeler, 1922; = *Hendecatella* Wheeler, 1927; = *Solenops* Karawajew, 1930; = *Sporocleptes* Arnold, 1948; = *Crateropsis* Patrizi, 1948; = *Nimbamyrma* Bernard, 1953; = *Afroxyidris* Belshaw & Bolton, 1994 (provisional); = *Neoblepharidatta* Sheela & Narendran, 1997. Fernández (2010) has added *Parvimyrma* Eguchi & Bui, 2007 to the synonymy.

There are anomalies, however, in the Fernández proposal which was based primarily on the American fauna. In particular, it does not gel with the contrasting dimorphism of the *Oligomyrmex* workers, with minors, ca. 1.0 mm in total length (TL), and majors, TL ca. 2.0–2.5 mm, coupled, where known, with queens of a similar general morphology to the major workers and no more than twice as long, TL ca. 5–6 mm or less and the *Carebara* *s.s.* which have monomorphic workers with TL ca. 2.0 mm and grossly enlarged queens, most with TL 15 mm plus. The *Carebara* *s.s.* queens also are morphologically greatly dissimilar to any *Oligomyrmex* queens.

The genus *Carebara* *sensu*Fernández (2004) was unknown from Arabia prior to the description of *Carebara arabica* (= *Oligomyrmex arabicus*) from Yemen by Collingwood and Van Harten 2001). Although the description of *Carebara arabica* might have been more explicit, it was based on major and minor workers, with drawings of both. Here, we give the first record of a *Carebara* species from Saudi Arabia based on the new species, *Carebara abuhurayri*.

## Measurements and indices

Measurements in mm and indices are as follows (Bolton 1987):

TLTotal Length; the outstretched length of the ant from the mandibular apex to the gastral apex.

HWHead Width; the maximum width of the head behind eyes in full face view.

HLHead Length; the maximum length of the head, excluding the mandibles.

CICephalic Index (HW x 100/HL).

SLScape Length, excluding basal neck.

SIScape Index (SL x 100/HW).

ELEye Length; the maximum diameter of the eye.

MLMesosoma Length; the length of the mesosoma (or alitrunk) in lateral view, from the point at which the pronotum meets the cervical shield to the posterior base of the propodeal lobes or teeth.

PRWPronotal width in dorsal view.

PLPetiole Length; the maximum length measured in dorsal view, from the anterior margin to the posterior margin.

PWPetiole Width; maximum width measured in dorsal view.

PPLPostpetiole Length; maximum length measured in dorsal view.

PPWPostpetiole Width; maximum width measured in dorsal view.

## Taxonomy

### Carebara abuhurayri

Sharaf & Aldawood sp. n.

urn:lsid:zoobank.org:act:CCB8586A-7665-49D1-8CD7-62EE4F77FC7B

http://species-id.net/wiki/Carebara_abuhurayri

Figs 1 12

#### Holotype worker.

TL 0.99, HL 0.39, HW 0.31, SL 0.24, ML 0.31, PRW 0.19, PL 0.11, PW 0.08, PPL 0.05, PPW 0.09, SI 77, CI 79.

**Figures 1–8.**
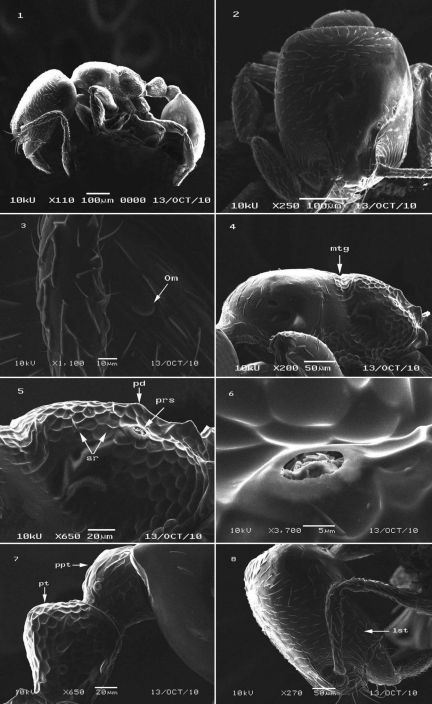
*Carebara abuhurayri* sp. n.; ar: areolate-rugose; lst: longitudinal striations; mtg: metanotal groove; om: ommatidiun; pd: propodeum; prs: propodeal spiracle; ppt: postpetiole; pt: petiole.

Overall unicolorous yellow, smooth and shining (Fig. 1). Head (Fig. 2) distinctly longer than broad, with clearly convex sides and a straight posterior margin. Mandibles smooth and shining with relatively long yellow hairs and armed with four teeth. Median portion of clypeus flat. In anterolateral view, clypeal lateral carinae strongly narrowed posteriorly between frontal lobes, then continued as a frontal triangle. Eyes minute and with a single ommatidium (Fig. 3).Scapes fail to reach head posterior margin by about one-third the head length. The scapes broaden evenly from about mid-length. Mesosoma in profile slightly convex. Metanotal groove shallow but distinct, dorsally and laterally (Fig. 4). Propodeum obliquely angled (Fig. 5). Propodeal spiracle (Fig. 5, 6) relatively large, circular, high and close to propodeal declivity. Metapleural gland orifice prominent. Petiole longer than broad in dorsal view with short peduncle. Postpetiole node lower than petiole and dorsally distinctly convex, nearly as long as broad in dorsal view (Fig. 7). Pilosity appressed, few and short on mesosoma, petiole, postpetiole, and rare on first gastral tergite, underside of head with a few short straight hairs. The clypeus has two pairs of standing hairs, the central pair long, and the lateral pair shorter. Anterior sides of head very finely longitudinally striated (Fig. 8). Dorsum of head with abundant scattered hair pits. Lower half of mesopleura, metapleura, and petiole and postpetiole with areolate-rugose sculpture (Fig. 5).

#### Paratypes.

 TL 0.99–1.13, HL 0.35–0.41, HW 0.29–0.32, SL 0.21–0.28, ML 0.31–0.34, PRW 0.17–0.19, PL 0.08–0.12, PW 0.07–0.08, PPL 0.05–0.07, PPW 0.08–09, SI 69–88, CI 74–89. (7 measured).

#### Holotype worker.

 Saudi Arabia, Al Bahah, Al Mukhwah, Zei Ein Archaeological Village, 19°55’N; 41°26’E, 741 m. a.s.l. 18.v.2010 (*M. R. Sharaf Leg.*); deposited in the King Saud Museum of Arthropods, College of Food and Agriculture Sciences, King Saud University, Riyadh, Kingdom of Saudi Arabia.

#### Paratypes.

7 workers, same localoty as holotype; 1 deposited in the Muséum ďHistoire Naturelle, Geneva, Switzerland (Dr Bernhard Merz); 1 deposited in Naturhistorisches Museum, Basel, Switzerland (Mrs. Isabelle Zürcher-Pfander); 1 deposited in California Academy of Science (Dr Brian Fisher); 2 deposited in World Museum Liverpool, Liverpool, U.K (Dr Guy Knight), the remaining specimens in the King Saud Museum of Arthropods, King Saud University, Riyadh, Saudi Arabia.

Given the anomalies of the Fernández (2004) schema and with only minute monomorphic workers, we are unable to place this new species within his species-complexes (denominated as species–groups in Fernández 2010). In the old schema, workers with 10-segmented antennae would fall in the Genus *Oligomyrmex* Mayr subgenus *Aeromyrma* Forel. Those, however, like all the *Oligomyrmex*, have dimorphic workers but, from the present collection, *Carebara abuhurayri*has only a small worker morph. *Carebara arabica* has major and minor workers, both appearing to have 11-segmented antennae. The minor is larger, TL 1.3 mm, than *Carebara abuhurayri*, TL max 1.13 mm, and the propodeum profile of the minor has a sharp angular transition from the dorsum to the declivity.

**Figures 9–12.**
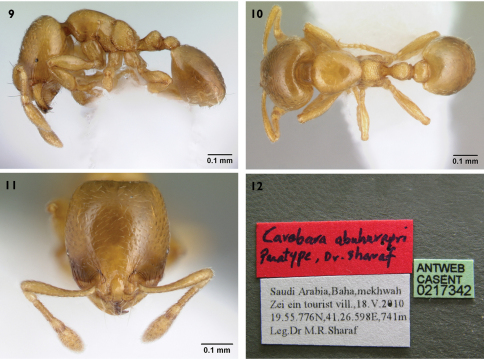
*Carebara abuhurayri* sp. n. paratype worker **9–12, 9** body in profile **10** body in dorsal view **11** head in full-face view **12** type locality label (CASC)

#### Etymology.

 This new species is named after Abuhurayra, the companion of the Prophet, Mohammed, may peace and blessing be upon him, and whose tribe inhabited Al Bahah region.

#### Biology.

 The specimens of *Carebara abuhurayri* were found foraging on the ground and coexisting with the ant species *Tetramorium sericeiventre* Emery, 1877, *Pheidole minuscula* Bernard, 1851, *Pheidole* sp., *Monomorium destructor* (Jerdon, 1851), *Monomorium exiguum* (Forel, 1894), *Monomorium* sp. and *Crematogaster* sp. This association with the above taxa may indicate a “lestobiotic” relationship (Longino, 2004) but at present, it is not known with which of these above species *Carebara abuhurayri* is nesting. It is worth mentioning that *Carebara abuhurayri* is one of the smallest ant species known to occur in Arabia.

**Figure 13.**
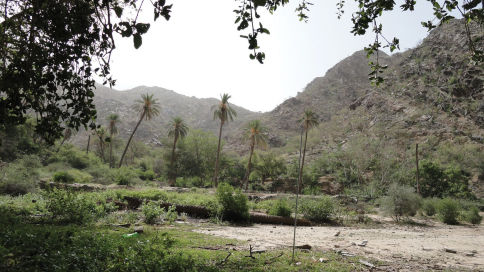
Type locality, Al Mukhwah, Zei Ein Archaeological village.

The type locality is a mountainous area which is considered as a part of upper Tihama territory which belongs to Al Bahah region (Fig. 13). The locality has a great diversity of wild plants and many cultivated fruits, especially banana, date palm, and *Ficus* trees, also alfalfa, and some lemon trees are cultivated. Many water streams are present in the area, therefore, the soil has a considerable degree of humidity all year round. Such habitats are found elsewhere in Arabia and so this or related species can be expected in most Arabian countries. For Saudi Arabia, we are expecting to record them in the Asir mountain chain, especially in the lower elevation areas which are called Tihama. We hope future collecting will allow clarification as to whether *Carebara abuhurayri* has monomorphic or dimorphic workers and the nature of the queen.

## Discussion

### Additional Arabian species

*Carebara arabica* (Collingwood & van Harten, 2001) which was described as *Oligomyrmex arabicus* from Yemen based on major and minor workers, and is known only from a single collection, is an example of a long-headed species with 11-segmented antennae (in the original description, the SL for major is given wrongly as 0.63, from the illustration it would be ca 0.16). The small worker of the new species *Carebara abuhurayri* appears not too dissimilar to the minor worker of *Carebara arabica* but it is consistently smaller in size (TL 0.99–1.13 mm versus TL 1.30); has a higher cephalic index (CI 74–89 versus CI 71), and a relatively lower head length (HL 0.35–0.41 versus HL 0.42). In addition, *Carebara abuhurayri* has a distinct but shallow metanotal groove compared with the deep groove in *Carebara arabica*. It does not resemble *Carebara afghanus* Pisarski, 1990, which has 9-segmented antennae but has a low, elongated and flat alitrunk profile without propodeal spines. The presence of single facet eyes, however, is the main characteristic, that sets *Carebara abuhurayri* apart from some of the African *Carebara*.

In pre-Fernández taxonomy *Carebara abuhurayri* might fall in the *Oligomyrmex* subgenus *Aeromyrma*, i.e. those with 10-segmented antennae. The only sub-Saharan species with 4-toothed mandibles is *Oligomyrmex jeanneli* Santschi, 1913. This has minor, TL 0.9 mm; metanotal groove shallow, dorsum of propodeum short; petiole noticeably narrower than postpetiole, postpetiole wider than long; head smooth, feebly punctuate, shiny; eyes atrophied set at anterior third of side; scape reaches posterior third of the head; petiole wider than high; postpetiole transverse, twice as wide as long; promesonotum wider than long; dorsum of propodeum wider than long unarmed; yellow, smooth and shiny.

## Supplementary Material

XML Treatment for Carebara abuhurayri
